# Endovascular Embolization of Ruptured Infundibular Dilation of Posterior Communicating Artery: A Case Report

**DOI:** 10.1155/2010/210397

**Published:** 2010-12-27

**Authors:** Jinlu Yu, Honglei Wang, Kan Xu, Bai Wang, Qi Luo

**Affiliations:** ^1^Department of Neurosurgery, First Hospital of Jilin University, Changchun 130021, China; ^2^Department of Radiology, First Hospital of Jilin University, Changchun 130021, China

## Abstract

Hemorrhage due to the rupture of the infundibular dilatation of the posterior communicating artery (ID of the PCo-A) occurs infrequently. The preferred treatment of such hemorrhages is surgical clipping through craniotomy. There are few reports about endovascular coil embolization in such cases. We report such a case treated by endovascular embolization. A 35-year-old man, who had experienced 2 episodes of subarachnoid hemorrhage (SAH), was found to have a ruptured ID of the PCo-A by head computed tomography angiography (CTA) and digital subtraction angiography (DSA). We performed stent-assisted endovascular coil embolization through a combined anterior and posterior circulation approach. Postembolization angiography showed absence of contrast filling of the ID of the PCo-A and nonleakage of the contrast agent. The patient recovered well with no complications. SAH recurrence was not recorded during the 1-year followup. The postoperative angiographic result was good. To our knowledge, this is the first case of hemorrhage due to ruptured ID of the PCo-A that was treated by such a technique.

## 1. Introduction

ID of the PCo-A refers to funnel-shaped widenings with a diameter less than 3 mm at the junction of the posterior communicating artery and the internal carotid artery [1–3]. ID of the PCo-A was regarded as normal anatomical variants with a low risk of rupture [4]. However, with increasing reports of ruptured ID of the PCo-A, bleeding is also found to occur in cases of ID of the PCo-A. Aggressive treatment (i.e., surgical clipping) is preferred for most patients presenting with hemorrhage [2–6], but little is known about the endovascular treatment of this condition. We report a case of ruptured ID of the PCo-A successfully treated by endovascular embolization.

## 2. Case Presentation

**Figure 1:**
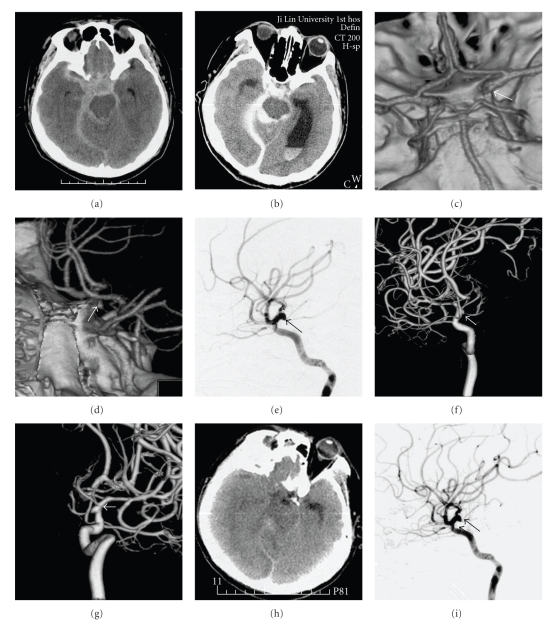
Diagnosis of ruptured ID of the PCo-A. (a, b) Two SAH images detected at an interval of 9 hours show that the bleeding points in both episodes of hemorrhage were centered around the origin of the right posterior communicating artery and that the suprasellar cistern, sylvian cistern, ambient cistern, and tentorial margin were involved. Blood entry into the ventricles and the presence of ventriculomegaly were seen in (b). (c, d) Head CTA showed that a funnel-shaped expansion with a diameter of less than 3 mm occurred at the origin of the right posterior communicating artery and was in conjunction with the thin right posterior communicating artery at its apex (arrow). (e)–(g) The first DSA showed that a funnel-shaped expansion occurred at the origin of the right posterior artery and was in conjunction with the thin right posterior communicating artery at its apex, a finding similar to the results of CTA (arrow). (h) Reexamination by head CT after 20 days of conservative treatment demonstrated that SAH was absorbed and the size of ventricles was normal. (i) The second DSA examination after 20 days of conservative treatment revealed that the funnel-shaped expansion still existed at the origin of the right posterior communicating artery and was in conjunction with the thin posterior communicating artery at its apex, similar to the results obtained by CTA and the first DSA (arrow).

A 35-year-old man was admitted with sudden onset of headache and vomiting. At admission, he was in relatively good condition. Hunt-Hess scale was grade III, and the GCS score was 13 at presentation. A CT scan of the head revealed SAH mainly in the right suprasellar cistern with a Fisher grade of 3 (Figure 1(a)). Dehydration therapy was performed immediately. Nine hours after the onset of symptoms, the patient lapsed into a coma; Hunt-Hess grade progressed to IV, and the GCS score deteriorated to 8. A repeated head CT examination revealed rebleeding, acute hydrocephalus, hematocele at bilateral posterior horns of the lateral ventricle, and a Fisher grade of 4 (Figure 1(b)). A CTA was performed together with a CT and revealed a conical expansion of the infundibulum at the origin of the right posterior communicating artery with a diameter of less than 3 mm. The thin right posterior communicating artery arose from the apex of the infundibulum. In addition, no aneurysm was noted in angiography (arrow in Figures 1(c) and 1(d)). DSA and 3-D reconstruction were then performed, and these findings were similar to those seen in the CTA (arrow in Figures 1(e), 1(f), and 1(g)). After these imagings, conservative treatments including dehydration therapy were administered to allow the reabsorption of SAH. Nimodipine was given for antivasospasm treatment. In the meantime, lumbar drainage was performed to drain bloody cerebrospinal fluid. 

After 20 days of conservative treatment, the patient's condition got better, and he could follow simple commands. A repeated head CT showed that SAH was reabsorbed, and the size of the ventricles became normal (Figure 1(h)). DSA was performed again and yielded the same result as the previous one (arrow in Figure 1(i)). Thus, ruptured ID of the PCo-A was diagnosed, and we planned to perform coil embolization. Because of the wide neck of the ID, stent-assisted occlusion was required. As a result, clopidogrel and aspirin were administrated orally for one week.

**Figure 2:**
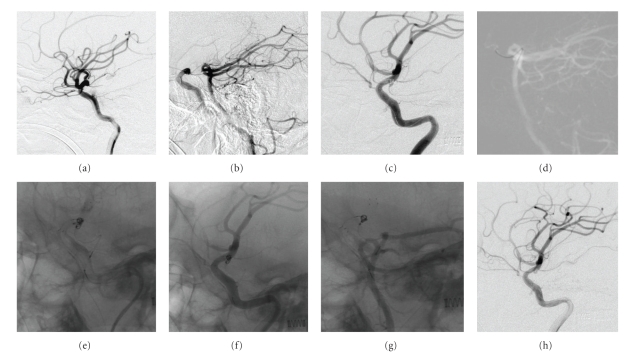
Endovascular coil embolization of ruptured ID of the PCo-A. (a) DSA before treatment showed that a funnel-shaped expansion with a diameter of 2 mm occurred at the origin of the right posterior communicating artery and was in conjunction with the posterior communicating artery at its apex. (b) Vertebral angiography showed that blood flew reversely from the posterior communicating artery into the internal carotid artery system after carotid artery compression. (c) A Neuroform3 stent was released in the internal carotid artery to cover the origin of the posterior communicating artery; an Echelon-10 microcatheter was used to release a Hydrocoil10 coil into the PCo-A ID through the stent mesh using a Transend300 microguidewire, but the coil was not detached. (d) A microcatheter was placed into ID of the PCo-A through the posterior vertebral artery and posterior communicating artery under the guidance of Roadmap. (e) Four Hydrocoil10 coils were released into ID of the PCo-A through anterior and posterior circulation. (f, g) No enhancement of ID of the PCo-A was noted after five coils had been filled into anterior and posterior circulation, and there was no contrast agent leakage. (h) Angiography of coils in anterior and posterior circulation after they were all detached, showing no enhancement of ID of the PCo-A and the posterior communicating artery.

At day 27 after the bleed, endovascular coil embolization was performed under general anesthesia. The right femoral artery was punctured using the Seldinger technique to introduce a vascular sheath. Heparin was administered. Vertebral angiography with carotid compression was performed before vascular embolization so as to observe the blood supply to the posterior circulation of the ID of the PCo-A. The result showed that blood flow reversed into the internal carotid artery through the posterior communicating artery (Figures 2(a) and 2(b)). Subsequently, endovascular embolization of the ID of the PCo-A was conducted. After a suitable working angle was selected, a Neuroform3 stent (4.0 mm × 20 mm) was successfully released using an Echelon-14 microcatheter and Transend-300 microguidewire to completely cover the neck of the ID of the PCo-A. Subsequently, an Echelon-10 microcatheter was delivered into the ID of the PCo-A using Transend-300 microguidewire through the stent mesh. After angiography, a HydroCoil 10 coil (2 mm × 2 cm, MicroVention, Aliso Viejo, Calif) was released into the ID of the PCo-A. However, the coil was not detached due to the unfavorable location (Figure 2(c)). The left femoral artery was punctured for introduction of a vascular sheath, and then an Echelon-10 microcatheter was placed into the PCo-A ID using a Transend-300 microguidewire via the posterior communicating artery (Figure 2(d)). Four HydroCoil 10 coils were sequentially released (2 mm × 2 cm, 2 mm × 2 cm, 2 mm ×1 cm, 2 mm × 1 cm) to fill the ID of the PCo-A (Figure 2(e)). No enhancement of the ID of the PCo-A was shown in the angiography of anterior or posterior circulation, and leakage of the contrast agent did not occur (Figures 2(f) and 2(g)). The coil placed in the internal carotid artery was detached (Figure 2(h)). After catheters and sheaths were removed, the procedure was completed.

**Figure 3:**
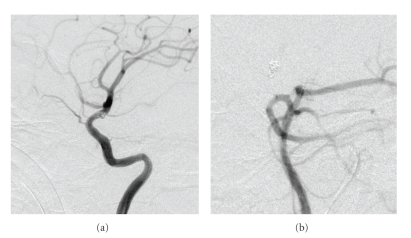
Follow-up DSA. (a, b) The follow-up DSA did not show the recurrence of ID of the PCo-A.

Postoperative course was uneventful. The patient was treated with clopidogrel and aspirin. During a 1-year followup, the patient recovered well and could care for himself. His GOS score was 4, and SAH did not recur. The follow-up DSA showed no signs of ID of the PCo-A (Figures 3(a) and 3(b)).

## 3. Discussion

ID of the PCo-A is not a rare clinical entity. However, the prevalence of it on carotid angiography ranges from 5% to 17% [6–9] or even higher, as shown in other studies [10, 11]. The natural history of ID of the PCo-A remains unclear; it is generally recognized as an anatomical variant resulting from incomplete retrogression of vessels during embryonic development. ID of the PCo-A usually does not rupture or bleed [6, 8]. However, a number of studies have reported fatal SAH caused by ID of the PCo-A rupture or its progression to aneurysms [3, 4]. Hemorrhage due to the rupture of the ID of the PCo-A is surgically treated (i.e., surgical clipping) to prevent disastrous consequences due to rebleeding [5, 6, 8, 12–20]. In most IDs of the PCo-A, PCo-A is not well developed and has few perforating branches; so, the origin of the ID of the PCo-A is clipped in an orientation parallel to the internal carotid artery to prevent rebleeding [12–18]. 

However, in our patient, although the right PCo-A was underdeveloped, neurosurgical clipping treatment was not selected, and stent-assisted endovascular coil embolization was performed using a combined approach of anterior and posterior circulation. This approach was considered because we found that the ID of the PCo-A had a wide junction with the internal carotid artery, making full clipping quite difficult similar to wide-necked aneurysms [21]. Moreover, a previous study has shown that the bleeding points in the ruptured ID of the PCo-A were mostly located at the distal side of the ID, close to the internal carotid artery [22]. Under such circumstances, blood could still flow into the inside of the ID along the PCo-A even when the origin of the ID is clipped. 

In the current study, enhancement of the PCo-A was not revealed in the DSA of posterior circulation, but it appeared in the angiography of the compressed internal carotid artery. Blood flowed reversely into the internal carotid artery (Figure 2(b)) indicating the possibility of rebleeding if the neck of the ID was clipped and the ruptured bleeding point was left untreated. However, clipping the bleeding points of the ID of the PCo-A might result in new bleeding sites near the previous ones, due to the continuous impact of blood flow; there is also the probability that the ID of the PCo-A will progress to an aneurysm [22, 23]. Therefore, at least 2 aneurysm clips are needed for isolation of the ID of the PCo-A in order to prevent rebleeding. It is difficult to place two aneurysm clips because of the narrow operating space around the posterior communicating artery. On the contrary, stent-assisted coil embolization by means of endovascular approach is relatively safe in comparison to neurosurgical clipping. Therefore, we preferred to perform endovascular treatment. 

Although the patient experienced two SAH episodes in this study, the CTA on admission and first the DSA did not reveal the aneurysm but a conical expansion of the infundibulum at the origin of the right posterior communicating artery. At that time, ruptured ID of PCo-A could not be confirmed because profound SAH might have resulted in vasospasms that hide a very small aneurysm. In addition, the condition of the patient was critical. If we had attempted to treat the expansion of the infundibulum, disastrous result might have occurred or the aneurysm might have been missed. The better alternative was to wait until the blood was reabsorbed. After 20 days of conservative treatment, the repeated CT revealed reabsorption of blood along with an improvement in the condition of the patient. The DSA was performed and confirmed the diagnosis of ruptured ID of the PCo-A. Endovascular treatment lead to a good outcome. The follow-up DSA did not show the recurrence of ID of the PCo-A.

To date, no report on endovascular intervention treatment for ruptured ID of the PCo-A is available. We made an attempt to provide a new strategy for its treatment. While considering endovascular embolization for eligible patients with ID of the PCo-A, the following two factors need to be taken into account. First, the ipsilateral internal carotid artery needs to be compressed when conducting vertebral angiography so as to fully assess the hemodynamic status of the ID of the PCo-A. Therefore, rupture due to incomplete filling and blood supply from the posterior circulation can be prevented. Second, stent-assisted embolization is required because of the wide neck of the ID of the PCo-A. When full blocking is not possible through the internal carotid artery of the anterior circulation alone, it is preferable to carry out the blocking through the combined approach of anterior and posterior (via the posterior communicating artery) circulation.

## 4. Conclusion

Stent-assisted endovascular coil embolization through a combined approach of anterior and posterior circulation provides a new strategy for treatment of hemorrhage due to rupture of the ID of the PCo-A.

## References

[1] Krayenbuhl HA, Yasargil M (1968). *Cerebral Angiography*.

[2] Saltzman GF (1959). Infundibular widening of the posterior communicating artery studied by carotid angiography. *Acta Radiologica*.

[3] Ebina K, Ohkuma H, Iwabuchi T (1986). An angiographic study of incidence and morphology of infundibular dilatation of the posterior communicating artery. *Neuroradiology*.

[4] Epstein F, Ransohoff J, Budzilovich GN (1970). The clinical significance of junctional dilatation of the posterior communicating artery. *Journal of Neurosurgery*.

[5] Ohyama T, Ohara S, Momma F (1994). Fatal subarachnoid hemorrhage due to ruptured infundibular widening of the posterior communicating artery—case report. *Neurologia Medico-Chirurgica*.

[6] Kuwahara S, Uga S, Mori K (2001). Successful treatment of a ruptured enlarged infundibular widening of the posterior communicating artery: case report. *Neurologia Medico-Chirurgica*.

[7] Hallacq P, Piotin M, Moret J (2002). Endovascular occlusion of the posterior cerebral artery for the treatment of P2 segment aneurysms: retrospective review of a 10-year series. *American Journal of Neuroradiology*.

[8] Coupe NJ, Athwal RK, Marshman LAG, Brydon HL (2007). Subarachnoid hemorrhage emanating from a ruptured infundibulum. Case report and literature review. *Surgical Neurology*.

[9] Osborn AG (1999). *Diagnostic Cerebral Angiography*.

[10] Ebina K, Ohkuma H, Iwabuchi T (1986). An angiographic study of incidence and morphology of infundibular dilatation of the posterior communicating artery. *Neuroradiology*.

[11] Saltzman GF (1959). Infundibular widening of the posterior communicating artery studied by carotid angiography. *Acta Radiologica*.

[12] Satoh T, Omi M, Ohsako C (2006). Differential diagnosis of the infundibular dilation and aneurysm of internal carotid artery: assessment with fusion imaging of 3D MR cisternography/angiography. *American Journal of Neuroradiology*.

[13] Marshman LAG, Ward PJ, Walter PH, Dossetor RS (1998). The progression of an infundibulum to aneurysm formation and rupture: case report and literature review. *Neurosurgery*.

[14] Cowan JA, Barkhoudarian G, Yang LJS, Thompson BG (2004). Progression of a posterior communicating artery infundibulum into an aneurysm in a patient with Alagille syndrome: case report. *Journal of Neurosurgery*.

[15] Endo S, Furuichi S, Takaba M, Hirashima Y, Nishijima M, Takaku A (1995). Clinical study of enlarged infundibular dilation of the origin of the posterior communicating artery. *Journal of Neurosurgery*.

[16] Konig A (1996). Clipping infundibula. *Journal of Neurosurgery*.

[17] Zager EL, Hackney DB (1996). Clipping infundibula. *Journal of Neurosurgery*.

[18] Yoshida M, Anegawa S, Moritaka K (1981). Significance of infundibular dilatation in unexplained subarachnoid hemorrhage. *Neurosurgery*.

[19] Buhk JH, Kallenberg K, Mohr A, Dechent P, Knauth M (2009). Evaluation of angiographic computed tomography in the follow-up after endovascular treatment of cerebral aneurysms—a comparative study with DSA and TOF-MRA. *European Radiology*.

[20] Jung JY, Kim YB, Lee JW, Huh SK, Lee KC (2006). Spontaneous subarachnoid haemorrhage with negative initial angiography: a review of 143 cases. *Journal of Clinical Neuroscience*.

[21] Tanaka Y, Kobayashi S, Hongo K, Tada T, Nagashima H, Kakizawa Y (2000). Intentional body clipping of wide-necked basilar artery bifurcation aneurysms. *Journal of Neurosurgery*.

[22] Baek H, Jayaraman MV, Karniadakis GE (2009). Wall shear stress and pressure distribution on aneurysms and infundibulae in the posterior communicating artery bifurcation. *Annals of Biomedical Engineering*.

[23] Beumer D, Delwel EJ, Kleinrensink GJ, Akouri S, Torres A, Krisht AF (2007). The perforator-free zone of the posterior communicating artery and its relevance in approaches to the interpeduncular cistern, especially the transcavernous approach: an anatomic study. *Neurosurgery*.

